# A Chinese herbal formula, Yi-Qi-Fu-Sheng, inhibits migration/invasion of colorectal cancer by down-regulating MMP-2/9 via inhibiting the activation of ERK/MAPK signaling pathways

**DOI:** 10.1186/1472-6882-13-65

**Published:** 2013-03-18

**Authors:** Wanli Deng, Hua Sui, Qiaolin Wang, Nana He, Chunyan Duan, Liang Han, Qi Li, Ming Lu, Shuqin Lv

**Affiliations:** 1 Oncology Department I of Traditional Chinese Medical Hospital of Xinjiang Uygur Autonomous Region, Xinjiang, 830000, China; 2 Department of Medical Oncology, Shuguang Hospital, Shanghai University of Traditional Chinese Medicine, Shanghai, 201203, China; 3 Interventional Cancer Institute of Integrative Medicine & Putuo Hospital, Shanghai University of Traditional Chinese Medicine, Shanghai, 200062, China

**Keywords:** Colorectal cancer, Invasion and migration, Metalloproteinase-2/9, Extracellular signal-regulated kinase, Yi-Qi-Fu-Sheng herbal formula

## Abstract

**Background:**

A Chinese herbal formula, Yi-Qi-Fu-Sheng (YQFS), has long been employed clinically to treat cancer patients. We aimed to determine its effectiveness as a treatment method for colorectal cancer. We investigated the therapeutic effects of YQFS on colorectal cancer, as well as the underlying mechanisms, which have not previously been explored.

**Methods:**

First, YQFS was extracted and chemically characterized. We then tested the effects of YQFS on proliferation and migration by MTT and transwell migration assays *in vitro*. Mouse xenograft models of colorectal cancer were established by inoculation with HCT-116 cells, and mice received one of three oral doses (200, 400 and 800 mg/kg/day) to evaluate the effects of YQFS extract. Metalloproteinase-2/9 (MMP-2/9) expression in mice was evaluated by gelatin zymography assay. Apoptosis was evaluated by flow cytometry (FCM) analysis *in vitro* and by TUNEL assay *in vivo*. ERK and p-ERK expression were evaluated by western blot analysis at the protein level *in vitro,* and by quantitative RT-PCR at mRNA level *in vivo*.

**Results:**

Our results show that YQFS significantly inhibits colorectal cancer cell proliferation and induces apoptosis and cell cycle arrest at the G_1−_ and S-phase in HCT-116 cells. Furthermore, YQFS effectively retards tumor cell migration and invasion by inhibiting metalloproteinase-2/9 (MMP-2/9) expression, both *in vitro* and *in vivo*. Moreover, YQFS had an inhibitory effect on tumor growth *in vivo*, and induced apoptosis through the inhibition of the ERK1/2 pathway both *in vitro* and *in vivo.*

**Conclusion:**

These findings demonstrate that YQFS extract has an anti-tumor effect in colorectal cancer, which could be attributed to ERK1/2-dependent inhibition of MMP-2/9 expression.

## Background

Colorectal cancer has an incidence of approximately 150,000 per year in the United States and is the third leading cause of cancer-related deaths in both men and women [1]. Although surgery, chemotherapy and radiotherapy have been the mainstay of colorectal cancer treatment, traditional Chinese medicine (TCM) has the advantage of reducing cancer therapy-induced toxicity and is a popular form of complementary and alternative medicine (CAM) in China [2]. In recent years, with increased popularity with patients in China, the modified classic formula has been shown to further minimize the side effects of surgery, radiation and chemotherapy [3], increase immune function [4] and improve survival [5].

Mitogen-activated protein kinases (MAPK), which belong to a large family of serine-threonine kinases, form major cell proliferation signaling pathways from the cell surface to the nucleus [6]. The ERK/MAPK pathway is one of the most important pathways for cell proliferation, and several key growth factors and proto-oncogenes transduce signals that promote growth and differentiation through this cascade [7]. Several lines of evidence suggest that the ERK/MAPK pathway, but not the JNK pathway or the p38 MAPK pathway, is a major regulator of cell proliferation in colorectal cancer [8]. During oncogenic transformation, colorectal cancer cells escape normal growth and differentiation control mechanisms and acquire the ability to invade surrounding tissues and organs. Activation of ERK1/2 culminates in the phosphorylation of transcription factors that regulate specific genes to increase cell proliferation and inhibit apoptosis [9].

Traditional Chinese prescriptions and formulae, which are based on TCM principles, have been identified as effective anti-cancer drugs in for example breast carcinoma [10], gastric cancer [11] and colorectal cancer [12]. YQFS, a Chinese herbal formula, is composed of six herbs: Dang-shen (Codonopsis pilosula), Bai-zhu (Atractylodes macrocephala), Fu-Ling (Poria), Gan-cao (Radix Glycytthizae), Rou-dou-kou (Myristica fragrans) and Ba-yue-zha (Fiveleaf Akebia fruit), in a ratio of 4:3:4:3:3:3. The components of the herbal formula are mostly derived from the classic formula “Si-Jun-Zi Tang” in TCM, which has a long history in treating deficiencies of the spleen and qi in cancer patients.

Recent clinical studies suggest that Si-Jun-Zi Tang herbal formula possesses anti-tumor and immune enhancing properties in colorectal cancer [13]. Modification of the Si-Jun-Zi Tang formula has been shown to have a wide range of immunopotentiating effects. As an example, administering two drugs, Rou-dou-kou and Ba-yue-zha, found in the Si-Jun-Zi Tang formula, exhibited anti-cancer effects and provided an advantage above using chemotherapy alone [14-16].

Although the anti-cancer effects of YQFS herbal formula have previously been demonstrated, the underlying mechanisms, in colorectal cancer, remain unknown. In this study, our objective was to elucidate the effect and the molecular mechanism(s) of the Chinese herbal formula, YQFS, in colorectal cancer cells.

## Methods

### Cell culture and reagents

Human colorectal cancer HCT-116 cells were obtained from the cell bank of the Chinese Academy of Sciences (CAS) and maintained in RPMI 1640 medium supplemented with 10% (v/v) heat-inactivated fetal calf serum, 2 mM glutamine, 100 units/ml penicillin, and 100 μg/ml streptomycin (Invitrogen, Carlsbad, CA) at 37°C in a 5% CO_2_ humidified atmosphere.

### Preparation of YQFS

All crude drugs (defined as a dried, unprocessed plant, and referring to one that was or is an official drug plant or the source of a refined drug substance) of YQFS were purchased from a local herbal medicine market (Xinjiang, China). All components were deposited at the herbarium located in the College of Pharmacy, Xinjiang Medical University. Briefly, Dang-shen, Bai-zhu, Fu-Ling, Gan-cao, Rou-dou-kou and Ba-yue-zha were mixed at a ratio of 4:3:4:3:3:3. YQFS extracts were prepared according to a previously validated method [17]. The mixture (300 g) was homogenized to a fine powder and then extracted twice in a reflux condenser for 2 h with 75% ethanol in a heated water-bath. The pooled extract was filtered to remove debris, and the ethanol was removed by rotary evaporation under reduced pressure. The concentrated extract was then dried by lyophilization to obtain the YQFS extract at a yield of 190 g. According to HPLC analysis of the YQFS extract, the ratio of crude drug to ethanol extract was 1 g/0.633 g. The extract was stored at 4°C, dissolved in distilled water and diluted with physiologic saline for the animal tests.

### MTT cell viability assays

Drug sensitivity was determined using the MTT assay. Briefly, cells were trypsinized and plated out into 96 well plates at a density of 3 × 10^3^ cells per well. Cells were cultured overnight and re-fed with fresh medium at various concentrations of YQFS for 24 h. Thereafter, 50 μl 3-(4,4-dimethylthiazol-2-yl)-2,5-diphenyltetrazolium bromide (MTT) (Sigma-Aldrich, St. Louis, MO) in PBS was added to each well, incubated for 4 h at 37°C and the formazan crystals that formed were dissolved in 150 μl dimethyl sulfoxide. The optical density was recorded at 570 nm on a microplate reader (Bio-Rad, Hercules, CA).

### Apoptosis assays

#### Apoptosis assay in vitro

Cells were seeded in 6-well plates (4 × 10^5^/well). After 24 h, three dose concentrations of YQFS (obtained from the result of YQFS IC10 in the MTT assay) were added. Flow cytometry was used to detect apoptosis by determining the relative amount of Annexin V-FITC-positive-PI-negative cells, as previously described. Unstained cells, cells stained with Annexin V-FITC alone, and cells stained with propidium iodide alone were used as controls. Singly stained cells were used to adjust electronic compensation on FL1 and FL2 channels.

#### Apoptosis assay in vivo

Dual staining for a-SMA and TUNEL was undertaken in representative liver sections to localize apoptotic hepatic stellate cells (HSCs). The sections were blocked with Dako double stain blocking solution for 3 min. The primary antibody for a-SMA, in a dilution of 1:1000, was added and incubated for 30 min at room temperature. Histofine labeled polymer (Nichirei, Tokyo, Japan) was added for 20 min at room temperature. The sections were then incubated with the substrate chromogen nitro-blue tetrazolium (NBT) for 5 min. The number of apoptotic HSCs were counted by viewing specimens under the microscope. The apoptosis index was defined as the number of apoptotic cells in every hundred cells counted.

#### Wound healing assay

Migration assays were performed following a standard protocol wound repair assay [18]. Briefly, cells were cultured in standard conditions, as described above, to 70%-80% confluency after treatment with YQFS. The monolayers were incubated and wounded in a line across the well with a standard pipette tip. The wounded monolayers were washed twice with phosphate-buffered saline (PBS) and incubated with serum-containing medium. The rate of wound closure was measured and photographed over 24 h. This allows imaging of both wound edges using the 10× objective.

#### Western blot analysis

Whole cell lysate for SDS-PAGE and western blot analysis for ERK expression was prepared as previously reported [19]. To prepare the lysates from dissected *in vivo* tumors, samples were snap frozen in liquid nitrogen immediately after sacrificing the animals and stored at −80°C. The lysate was incubated on ice in immunoprecipitation assay buffer for 2 h before being homogenized using a mortar and pestle. The homogenized sample was centrifuged and the supernatant was collected and stored at −80°C. Protein quantification and western blotting for ERK were done as previously reported [19]. Equal loading was confirmed with β-actin (0.1 μg/mL, Sigma Chemical). Densitometric analysis was done using Scion Imaging software (Scion Corporation), using total ERK or β-actin as a control for each sample.

#### Animals

##### Tumor xenograft animal model

Male athymic nude mice (NCr-nu) were purchased from Sino-British SIPPR/BK lab Animal Ltd., Co (Shanghai, China, license No. SCXK 2010–0002) and maintained under specific pathogen-free conditions for the studies. All animal protocols were approved by the Institutional Animal Use and Care Committee. All experiments and animal care protocols were approved by the Shanghai Medical Experimental Animal Care Commission in accordance with the Provision and General Recommendation of Chinese Experimental Animals Administration Legislation. The mice used in these experiments were 8–12 weeks old.

#### Drug administration

HCT116 cells were grown in culture and then detached by trypsinization, washed, and resuspended in HBSS. Next, 0.2 mL of the resuspended cells (1.0 × 10^6^) were subcutaneously injected into the athymic nude mice to initiate tumor growth. Tumors were allowed to reach an average size of 100 mm^3^, after which mice were randomized into 4 groups (n = 10 per group). Mice in group 1 were administered distilled water daily, which served as a vehicle control. Mice in group 2 were given 5-fluorouracil (5-FU) intraperitoneally, every 2 days, at a dosage of 0.5 mg/kg, half the maximum tolerated dose (MTD) of 5-FU, as previously described [20]. Mice in groups 3,4 and 5 received YQFS at a daily dose of 200, 400 or 800 mg/kg respectively, by intragastric administration for 19 days. In the clinical practice of Chinese herbal medicine, YQFS is usually prescribed at a daily dose of 400 mg herbal materials. When this human dose was converted into an animal dose (at an extraction yield of 2.5%, for a person weighing 60 kg, and a conversion factor of 12.33 between humans and mice), it was equivalent to the middle dose (400 mg extract/kg) used in this study.

Body weight and tumor growth were measured every 2 days. Tumor growth was determined by measuring the major (L) and minor (W) diameter with a caliper. The tumor volume was calculated according to the following formula: tumor volume = π/6 × L × W^2^. At the end of experiment, the animals were anesthetized with pentobarbital, and the tumor tissue was removed and weighed.

#### Gelatin zymography of MMP-2/9 activity

All tumor groups were plated onto 12-well culture plates and made quiescent by incubation in serum-free DMEM/F-12 for 24 h. The culture medium was collected and centrifuged at 10 000 × *g* for 5 min at 4°C to remove cell debris. MMP-2/9 expression was analyzed as previously described [9]. The gel analysis function in ImageJ (http://rsbweb.nih.gov/ij/) was used to quantify protease activity bands by densitometry. Values are reported in Relative Intensity Units (RIU).

#### Quantitative RT-PCR analysis

For RNA isolation, tumors were homogenized and suspended, and RNA was extracted using the RNAspin Mini Kit (GE Healthcare, Waukesha, WI, USA) according to the manufacturer’s instructions. For cDNA synthesis, 1 μg of total RNA was reverse-transcribed using oligo-dT primers and the Superscript Amplification System (Life Technologies, Carlsbad, CA, USA). Quantitative RT-PCR was carried out using SYBR Green PCR Master Mix (Life Technologies). The PCR amplification program consisted of an initial polymerase activation at 94°C for 5 min, followed by 35 cycles at 94 C for 30 s, 59.5°C for 40 s and 72 C for 30 s for VEGF. Amplification of GAPDGH, a relatively invariant internal reference RNA, was performed in parallel, and cDNA amounts were standardized to equivalent GAPDGH mRNA levels. Oligonucleotide primers for VEGF and GAPDGH were as follows: Oligonucleotide sequence (5’-3’) of ERK1/2 (188 bp), F: 5’-CCTGCTGGACCGGATGTTA-3’, R: 5’-GTCTCTTGGAAGATCAGCTC-3’, Oligonucleotide sequence (5’-3’) of GAPDH (306 bp), F: 5’-ACCCACTCCTCCACCTTTGA-3’, R: 5’-CTGTTGCTGTAGCCAAATTCGT-3’. mRNA expression was determined by real-time PCR using the TaqMan method as previously described [19].

#### Statistical analysis

All values were expressed as the mean ± S.D. and analyzed by one-way analysis of variance (ANOVA) followed by Duncan’s Multiple Range Test using SPSS version 13.0 software; a *p*-value of less than 0.05 was considered significant.

## Results

### Effect of YQFS on the viability of HCT-116 cells

**Figure 1:**
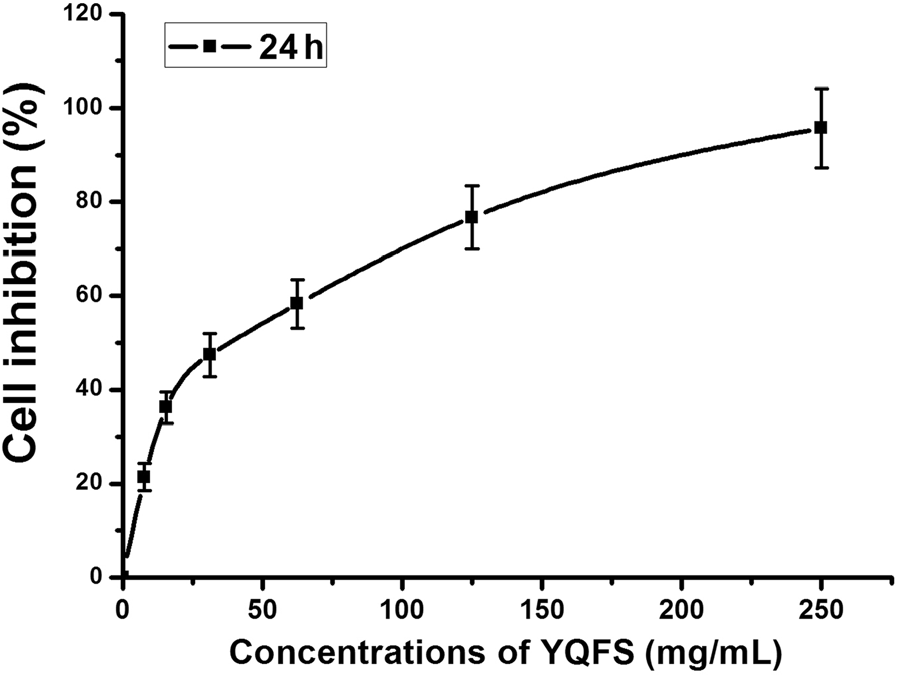
**Effect of YQFS on the inhibition of HCT-116 cells.** Cells were treated with various concentrations of YQFS and analyzed by MTT assay. The IC_50_ value is 34.09 mg/ml. For the dose–response analysis, we chose three doses below the IC_50_ value, and turned into a multiple relationship.

We used a simple and accurate HPLC method for the simultaneous separation and determination of six components to evaluate the quality of YQFS. We then tested their effects on the growth of the HCT-116 human colorectal cells *in vitro*. As shown in Figure 1, after treatment for 24 h, YQFS induced a dose-dependent decrease in cell viability in HCT-116 cells, as analyzed by MTT assay. HCT-116 cells showed a significantly reduced rate of cell growth.

### Effect of YQFS on apoptosis and cell cycle arrest at G_1_- and S-phase in HCT-116 cells

**Figure 2:**
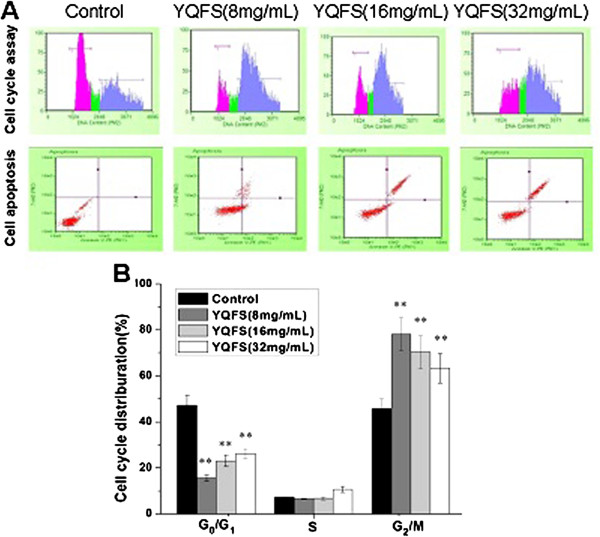
**The Effect of YQFS on apoptosis and the cell cycle in HCT-116 cells.** (**A**) Flow cytometry analysis of the cell cycle distribution of cultured HCT-116 cells with PI staining following 24 h exposure of cells to YQFS. Detection of apoptosis with Annexin V-FITC/PI binding to HCT-116 cells. Viable cells (Annexin V^−^/PI^−^) are located in the lower left, apoptotic cells (Annexin V^+^/PI^−^) in the lower right, post apoptotic secondary necrotic cells (Annexin V^+^/PI^+^) in the upper right and primary necrotic cells (Annexin V^−^/PI^+^) in the upper left quadrants, respectively. The numbers in each quadrant are the percentages of cells in question. (**B**) Distribution of HCT-116 cells’ cycle indicated by PI staining. The values shown are the mean ± SEM of 3 experiments. **P < 0.01 *vs.* the control.

We then tested whether YQFS affected cell cycle progression. The results showed a significant decrease in the number of cells in the proliferative G_1_- and S-phases and a significant increase in the number of cells in the G_2_-phase after 24 h of treatment with YQFS (Figure 2A). These results indicate cell cycle arrest at the S-phase after treatment of HCT-116 cells with YQFS. To confirm these results, we evaluated the effects of YQFS on apoptosis in HCT-116 cells. We observed a marked increase in both early and late stage apoptosis, as assessed by flow cytometry, in HCT-116 cells after YQFS treatment compared with control cells (Figure 2A). We then evaluated the effects of YQFS on apoptosis in HCT-116 cells by using annexin V-FITC and PI staining. Again, we saw a marked dose-dependent increase in both the early and late stage apoptosis, as assessed by flow cytometry, in HCT-116 cells after YQFS treatment, compared with control cells (Figure 2B).

### Effect of YQFS on migration measurement via the wound healing assay in HCT-116 cells

**Figure 3:**
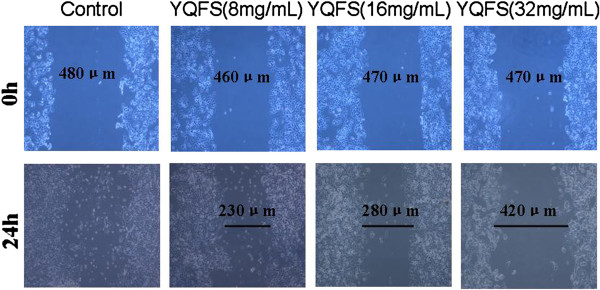
**Effect of YQFS on cell migration in a scratch wound repair assay.** Scratch-wound motility assays were carried out to detect wounded monolayers in HCT-116 cells. The effects of various concentrations of YQFS (8 mg/ml, 16 mg/ml and 32 mg/ml) on the migration of human aortic smooth muscle cells (HASMCs) at 0 h (upper row of images) and 24 h (lower row of images). Cell migration increased in the control and this effect was attenuated with the addition of YQFS.

We examined whether YQFS extract attenuated the motility of HCT-116 cells using the scratch wound repair assay. Cell migration increased after 24 h for the control group, but was substantially reduced when lower dose YQFS exerts was present (Figure 3). We also tested the effect of middle and higher dose of YQFS extract, the result showed a 40%-60% delay in wound closure after treatment for 24 h (Figure 3). These results suggest that YQFS inhibited migration/invasion in human colorectal cancer HCT-116 cells in a dose-dependent manner.

### Effect of YQFS on ERK phosphorylation in HCT-116 cells

**Figure 4:**
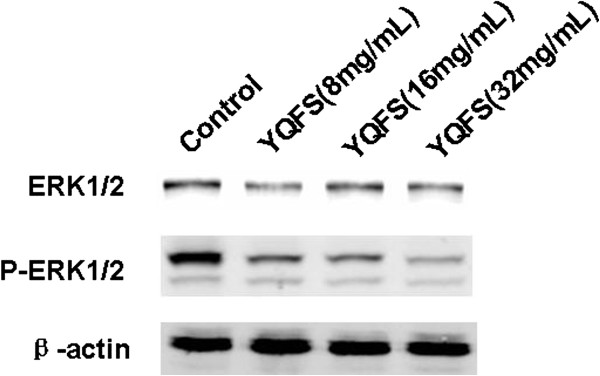
**The effect of YQFS on the expression of ERK and p-ERK in HCT-116 cells.** Western blotting assays were carried out to detect the activation of ERK in HCT-116 cells. HCT-116 cells were treated with YQFS at 8 mg/ml, 16 mg/ml and 32 mg/ml for 24 h respectively. Western blotting with was performed with an anti-β-actin antibody to ensure equal loading of proteins in each lane. The bolts were photographed and quantitated for each sample and the data are from three independent experiments.

To determine whether the MAPK pathways are involved in the anti-invasion and metastasis of tumor progression, the expression of JNK, ERK and p38 phosphorylation was examined in HCT-116 cells by western blotting. The results showed that the ERK signal transduction pathway was activated in HCT-116 cells. We observed decreased phosphorylation of ERK (p-ERK) in HCT-116 cells (Figure 4), but saw no significant effect in the levels of p-JNK or p-p38 (data not shown) after 24 h of YQFS treatment. These observations suggest that ERK, but not JNK or the p38/MAPK pathway, was inhibited by YQFS thus mediating anti-migration/invasion in human colorectal cancer HCT-116 cells.

### Effect of YQFS on tumor growth in colorectal cancer xenograft mice

In light of our *in vitro* data on the effect of YQFS in sensitizing colorectal cancer cells, we examined the therapeutic potential of YQFS *in vivo*. Based on our clinical data and preliminary experiments, mice were administered 12.33 times the dose given to human patients. Administration with herbal mixtures did not cause weight loss, decreased activity, or changes in fur quality of the rabbits (data not shown).

**Figure 5:**
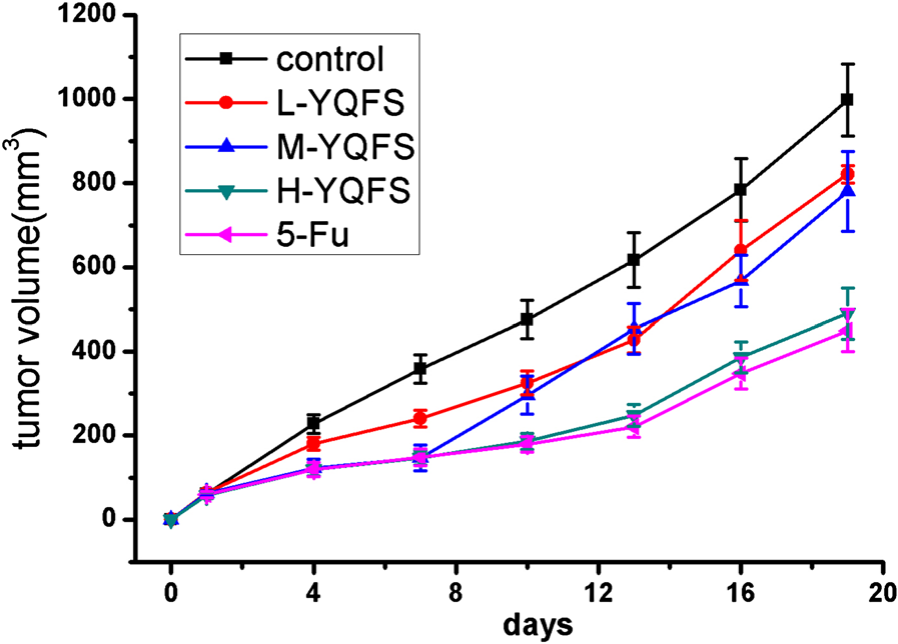
**The effect of YQFS on tumor growth *in vivo.*** After mice were injected with HCT-116 cells (2 × 10^7^), they were divided into five groups: Vehicle, L-YQFS (200 mg/kg), M-YQFS (400 mg/kg), and H-YQFS (800 mg/kg) were administered orally, each day for 18 days. The 5-Fu group was injected 5-Fluorouracil once every 2 days. Values were presented as mean ± SD., (n = 10).

The *in vivo* anti-tumor effect of YQFS was evaluated by measuring tumor weight and volume in colorectal cancer xenograft mice, while adverse effects were determined by measuring any body weight gain. As shown in Figure 5, H-YQFS treatment resulted in a 51% decrease in tumor volume compared with the control (997 ± 85 mm^3^ or 450 ± 49 respectively). Consistent with the tumor volumes, there was a 54% decrease in the tumor weights per mouse in the H-YQFS-treated group compared with the control group (1.06 ± 0.09 or 2.29 ± 0.30 grams respectively) as seen in Table 1. These suppressive effects of YQFS were dose-dependent. However, administration of different doses of YQFS (L-YQFS, M-YQFS and H-YQFS) had no effect on body weight gain in experimental animals (Table 1). Taken together, this suggests that YQFS is potent suppressor of colorectal tumor growth *in vivo*, with no apparent signs of toxicity.

**Table 1:** The effects of YQFS on body- and tumor weight of experimental animals (means ± SD)

**Group**	**Doses (mg/kg)**	**n**	**Body weight(g)**		**Tumor weight(g)**
			**Initial**	**Final**	
Control	-	10	20.00 ± 1.00	24.00 ± 1.00	2.29 ± 0.30
L- YQFS	200	10	18.00 ± 1.00	23.00 ± 0.50	1.88 ± 0.14
M- YQFS	400	10	19.00 ± 1.00	23.00 ± 1.00	1.46 ± 0.11*
H- YQFS	800	10	20.00 ± 1.00	24.00 ± 0.50	1.06 ± 0.09**
5-Fu	0.5	10	21.00 ± 1.00	23.40 ± 2.00	0.99 ± 0.08**

*P < 0.05 vs. control; **P < 0.01, vs. control.

### Effect of YQFS on apoptosis in colorectal cancer xenograft mice

**Figure 6:**
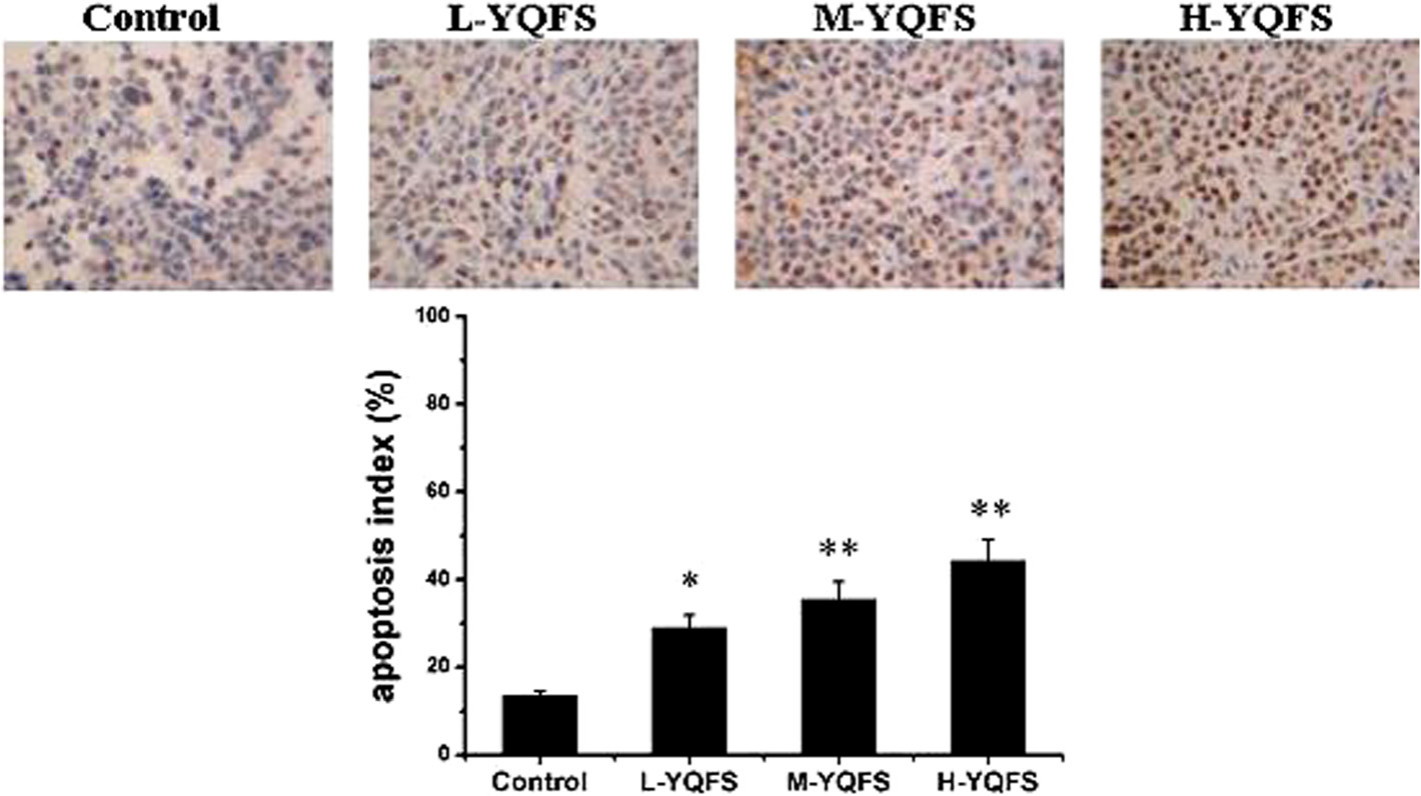
**The effect of YQFS on apoptosis *in vivo*.** A TUNEL assay was performed to detect apoptosis *in vivo* as described in the materials and methods section. The relative fold increase in apoptosis index is plotted as a graph. Each value is presented as the mean ± SD as determined from three independent experiments. *P < 0.05 *vs.* the control group; **P < 0.01 *vs.* the control group.

To determine whether the inhibitory effect of YQFS on cancer growth is related to cell proliferation and apoptosis, we examined the YQFS pro-apoptotic and anti-proliferative activities in colorectal cancer mice via immunohistochemical (IHC) staining for TUNEL and PCNA. The data in Figure 6 shows that the apoptotic index for H-YQFS was 44.24 ± 4.84%, 3.29 times that of the control (13.45 ± 1.09). These results are in agreement with our *in vitro* apoptosis results, which showed that YQFS significantly induced HCT-116 cell apoptosis in a dose-dependent manner. These data demonstrated that YQFS inhibited the proliferation of colorectal cancer cells and promoted cell apoptosis *in vivo*.

### Effect of YQFS on the expression of MMP-2/9

**Figure 7:**
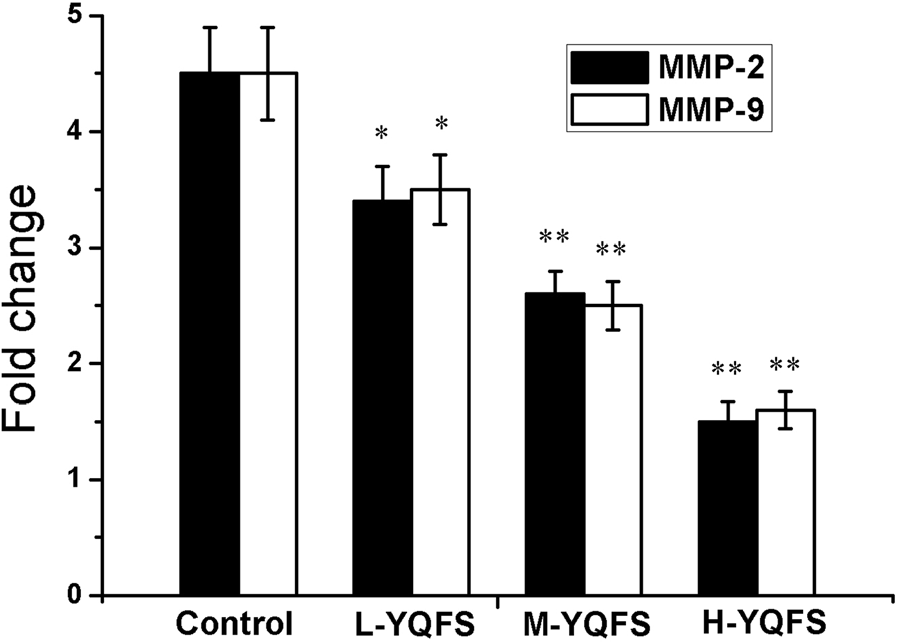
**The Effect of YQFS on the expression of MMP-2/9 *in vivo*.** Gelatin Zymography analysis of MMP-2/9 *in vivo,* as described in the materials and methods section. The relative fold change in expression of MMP-2/9 is plotted as a graph. Each value is presented as the mean ± SD as determined from three independent experiments. *P < 0.05 *vs.* the control; **P < 0.01 *vs.* the control.

Based on the preliminary experiments *in vitro*, we examined the expression of the angiogenic factors, MMP-2 and MMP-9, in tumors harvested from the various therapy groups (Figure 7). Both MMP-2 and MMP-9 expression was significantly lower in the YQFS-based therapy groups, suggesting that its antitumor effect may be mediated, in part, by downregulation of MMP-2/9 expression.

### Effect of YQFS on ERK phosphorylation in colorectal cancer xenograft mice

**Figure 8:**
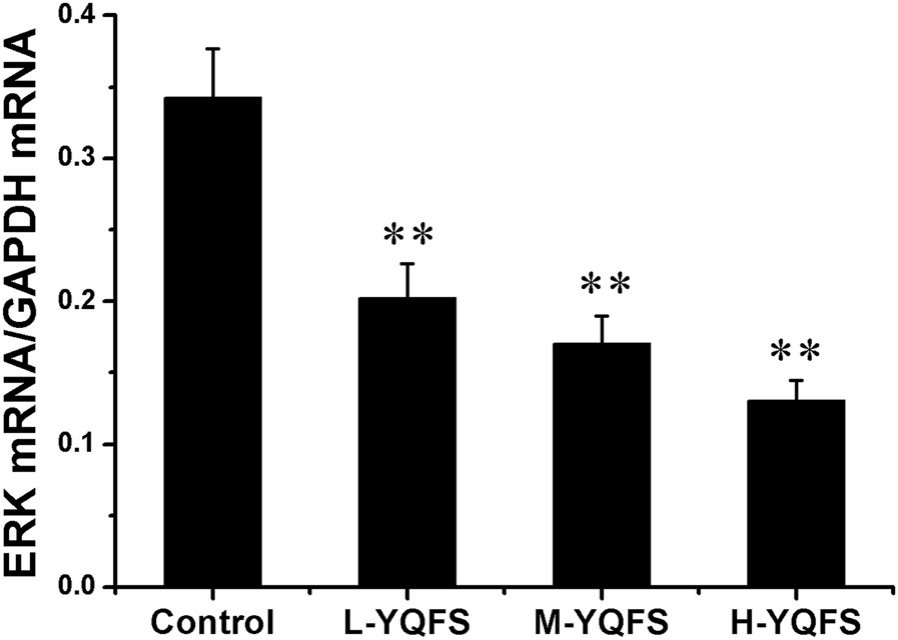
**The effect of YQFS on ERK mRNA levels *in vivo*.** Real-time quantitative PCR was performed to detect ERK mRNA *in vivo*. The data represented here were calculated from the means ± SD from triplicate experiments. **P < 0.01, *vs.* the control group.

Based on the preliminary experiments *in vitro*, we therefore examined the effect of YQFS on ERK mRNA levels in tumor tissue using quantitative RT-PCR. As shown in Figure 8, YQFS decreased ERK mRNA levels in a dose-dependent manner in mice tumors, with H-YQFS treatment resulting in a threefold decrease in ERK mRNA levels with respect to the control. The results shown are similar to that of MMP-2/9.

## Discussion

In this study, we demonstrated for the first time that YQFS inhibits tumorigenesis and cancer metastasis in colorectal cancer both *in vitro* and *in vivo*. Our results indicate that YQFS can modulate the ERK/MAPK pathway, and its downstream factors, by targeting ERK phosphorylation. We show that the growth of colorectal cancer cells is repressed by YQFS-induced apoptosis and inhibition of migration/invasion. *In vivo*, YQFS induced a significant delay in tumor growth, which could be mediated by decreased expression of MMP-2/9 via the ERK pathway. Our research therefore suggests that this Chinese Herbal medicine, YQFS, could inhibit migration/invasion of colorectal cancer by down-regulating MMP-2/9 via inhibition of the ERK/MAPK signaling pathway. YQFS therefore has previously undiscovered therapeutic potential for treating colorectal cancer.

In Asia, the incidence of colorectal cancer has become a national public health problem [21,22]. Despite advances in surgical and chemotherapeutic approaches, the resistance of colorectal cancer cells to traditional cytotoxic agents is a major obstacle in clinical cancer therapy, with most colorectal cancer patients eventually succumbing to the disease [12]. Therefore, novel therapeutic approaches for colorectal cancer are needed.

With the use of Traditional Chinese prescriptions and formulae, which are based on TCM principles, these agents have been identified as effective anti-cancer drugs in cancer patients, such as breast carcinoma, gastric cancer and colorectal cancer. Our previous clinical studies have demonstrated that YQFS prolongs the overall survival for stage IV colorectal cancer patients. It is now clear that administering herbal extracts may improve therapeutic outcomes among cancer patients. We therefore conducted a series of experiments to examine the potential mechanisms responsible for the therapeutic effects of YQFS.

To quantitate the effects of YQFS on cell growth, cell viability was assayed by reduction of MTT for 24 h with various concentrations of nimbolide. We found that YQFS treatment inhibited growth in colorectal cancer HCT-116 cells in a dose-dependent manner (Figure 1). We then investigated whether YQFS-induced inhibition of cell growth was due to perturbation of the cell cycle. Cell phase distribution analysis showed an accumulation of cells in the S-phase upon treatment with YQFS (Figure 2B), indicating that YQFS inhibits tumor cell proliferation and exerts its growth inhibitory actions through alterations in the cell cycle, and by inducing apoptosis.

It is well accepted that invasion and metastasis are integral parts of tumor progression and, investigators have reported that Physalis angulata markedly inhibits migration and invasion of highly metastatic HSC-3 cells, as shown by wound-healing repair- and trans-well-assays [23]. In our study, HCT-116 cell lines showed slower migration compared with the control after treatment with YQFS, judged by a scratch-wound motility assay (Figure 3). These data suggest that YQFS may modulate colorectal cancer invasion via altered cell motility.

The MAPK pathway represents one of the signaling cascades that link external stimuli to the transcriptional regulation of various molecules including those related to tumor-invasion and -metastasis. In mammals, three major MAPK pathways have been identified: extracellular-signal-regulated kinase (ERK), c-Jun N-terminal kinase (JNK), and p38 mitogen-activated protein kinase (p38 MAPK) [24]. There is growing evidence to support a strong correlation between ERK and invasive and metastatic potential in colorectal cancer [25]. In this study, we found that the increased levels of phosphorylated ERK1/2 in colorectal cancer HCT-116 cells were inhibited by YQFS in a dose-dependent manner. We also found that p38 and JNK1/2 were activated in colorectal cancer HCT-116 cells even though their activation levels differed. However, YQFS failed to inhibit phosphorylation of p38 and JNK1/2 in HCT-116 cells suggesting that YQFS might selectively target the ERK signaling pathway.

To further investigate the general effect of YQFS on colorectal cancer, we examined the effect of YQFS treatment *in vivo*. Our results are consistent with previous *in vivo* studies, where YQFS inhibited colorectal tumor growth compared with the control group in a dose-dependent manner. Furthermore, we found that chronic treatment with YQFS induced a significant increase in apoptosis in colorectal tumors, as judged by TUNEL staining. These results are consistent with studies performed by Gexia-Zhuyu Tang who showed that HSC apoptosis was induced via caspase 3 [26].

Within the past few years, many researchers have demonstrated the importance of MMPs in malignant transformation. MMP activity has been implicated in almost every stage of the metastatic cascade from the primary site to the progression of tumor-extravasation,- growth and -development. MMPs are enzymes that degrade structural components of the extra cellular matrix. These enzymes regulate a multitude of physiological processes such as morphogenesis, tissue remodeling and signaling events. Numerous studies have demonstrated a strong correlation between MMP-2/9 expression and migration/invasion in human colorectal cancer cells. In this study, we found a downregulation in MMP-2/9 expression following exposure to YQFS in rat colorectal tumors. These results are consistent with previous studies that evaluated the effects of MMP-2/9 inhibition on the suppression of tumor migration/invasion [27].

In recent years, there is increasing evidence to support the role of MMPs in mediating many changes in the tumor microenvironment during malignant transformation. Importantly, MAP kinase signaling such as ERK, JNK, p38, plays a significant role in MMP regulation, particularly MMP-2/9 in tumor cell migration. In this study, we observed that activation of the ERK pathway correlated with an increased expression of MMP-2/9. However, YQFS significantly reduced levels of p-ERK thus inhibiting the ERK pathway.

YQFS is the standard formulation of Si-Jun-Zi-Tang with the addition of Rou-dou-kou (Myristica fragrans) and Ba-yue-zha (Fiveleaf Akebia fruit) to create another formula known as Yi-Qi-Fu-Sheng-Fang. It was recently reported that Rou-dou-kou (Myristica fragrans), had a significant inhibitory effect on the growth of a colorectal cancer cell line [14]. Furthermore, Ba-yue-zha (Fiveleaf Akebia fruit), has been shown to remove toxic materials to inhibit tumor growth, and activate blood circulation to dissipate blood stasis [15,16], and to improve patients’ survival quality in 60 cases of intermediate and advanced malignant tumors [28]. In addition, previous studies have shown that Si-Jun-Zi-Tang can lessen the degree of post-operational stress and inflammatory response, and enhance the immune function of patients during enteral nutritional therapy [29]. *In vivo* studies have shown that SJZD can prevent indomethacin-induced damage of intestinal epithelial cells by inhibiting migration and proliferation of IEC-6 cells [30]. These results therefore strongly support the protective effects of YQFS against human colorectal cancer.

## Conclusions

In conclusion, the present study served to confirm the antitumor effects of YQFS *in vitro* and *in vivo*. Our results indicate that the therapeutic effect of YQFS may be explained by the inhibition of ERK-dependent migration/invasion. In addition, clarification of the underlying mechanism of YQFS will provide new anti-cancer therapeutic targets.

## Competing interest

The authors declare that they have no competing interests.

## Authors’ contributions

WLD and ML carried out the study and designed the experiments. QIW, NNH, and CYD contributed reagents, materials, and analysis tools. LH analyzed data. HS contributed the animal model. All authors read and approved the final manuscript.

## Pre-publication history

The pre-publication history for this paper can be accessed here:

http://www.biomedcentral.com/1472-6882/13/65/prepub
